# The mediating effect of work–life interference on the relationship between work-time control and depressive and musculoskeletal symptoms

**DOI:** 10.5271/sjweh.3887

**Published:** 2020-09-01

**Authors:** Sophie C Albrecht, Göran Kecklund, Constanze Leineweber

**Affiliations:** 1 Stress Research Institute, Stockholm University, Stockholm, Sweden

**Keywords:** autonomy, flexible work hour, psychosocial factor, working hour, work–life balance, mental health, depression, depressive symptom, musculoskeletal pain, physical health, cohort study, mediation, Swedish Longitudinal Occupational Survey of Health

## Abstract

**Objectives:**

Evidence shows that work-time control (WTC) affects health but underlying mechanisms are still unclear. Work–life interference (WLI) might be a step on the causal pathway. The present study examined whether WLI mediates effects on mental and physical health and contrasted these to other causal pathways.

**Methods:**

Four biennial waves from the Swedish Longitudinal Occupational Survey of Health (SLOSH, N=26 804) were used. Cross-lagged analyses were conducted to estimate if WLI mediated effects from WTC (differentiating between control over daily hours and time off) to subsequent depressive and musculoskeletal symptoms. Other causal directions (reversed mediation, direct and reversed direct effects) and robustness of mediation (by including covariates) were examined.

**Results:**

WLI partially mediated the relationship of WTC (control over daily hours/time off) with both health outcomes. Indirect effect estimates were small for depressive symptoms (-0.053 for control over time off and -0.018 for control over daily hours) and very small for musculoskeletal symptoms (-0.007 and -0.003, respectively). While other causal directions were generally weaker than causal mediational pathways, they played a larger role for musculoskeletal compared to depressive symptoms. Estimates relating to control over time off were in general larger than for control over daily hours.

**Conclusions:**

Our results suggest that WLI mediates part of the effect from WTC to mental/musculoskeletal symptoms, but small estimates suggest that (i) WTC plays a small but consistent role in effects on health and (ii) particularly regarding musculoskeletal disorders, other causal directions and mediators need to be further examined.

An increasing body of research presents evidence that workers’ control over their working hours – work-time control (WTC) – is associated with health outcomes over time (1–3). Adapted from Knauth (4), WTC can be described as an individual’s autonomy regarding duration and distribution of working hours. This definition points to a two-dimensional structure of WTC (5, 6): While control over daily working hours reflects daily length and starting and ending times of work, control over time off relates to taking time off from work in the form of taking breaks, running private errands during work, and scheduling vacation and other types of leave. Although WTC enables workers to structure working hours, this inevitably also affects non-work-time (7). WTC is conceptually related but not identical to the job-control dimension in the Job–Demands–Control Model (8). Job control refers to autonomy regarding content of work and how work tasks are performed, whereas WTC describes the temporal aspect of autonomy at work (9).

By allowing workers greater control over their working hours, positive effects on health, work–life balance, well-being and even performance are predicted. Evidence shows that while lower levels of WTC are associated with deteriorating health outcomes over time (10, 11), high WTC may prevent ill-health. In several longitudinal studies, higher levels of WTC were associated with fewer depressive symptoms, lower levels of fatigue, and lower risk of disability pension due to musculoskeletal and mental disorders (3, 12, 13). Some studies observed stronger effects of control over time off on health (in contrast to control over daily hours) suggesting that taking breaks and scheduling vacation is particularly important for workers (3, 14, 15). These results highlight that both mental and physical health are related to WTC, but the underlying mechanisms behind these associations are still not well understood.

On the one hand, effects of WTC on health can be explained by the effort–recovery model (16) stating that efforts spent at work need to be balanced with recovery time. Insufficient recovery can lead to adverse health outcomes. WTC allows to manage workload from a temporal perspective (control over daily hours) as well as ensure workers can take opportunities to recover both in and outside of working hours (control over time off) (1). On the other hand, WTC enables workers to manage (and reduce) strain due to conflicting responsibilities stemming from work and private/family life. By adapting working hours and time off from work, time between work and personal interests can be better balanced—which in turn affects health and well-being (1). To advance our understanding of causal pathways and mediator variables linking WTC with health, the present paper focused on the mediating role that work–family conflict may play in this relationship.

Work–family conflict can emerge when workers experience difficulties in balancing responsibilities from work and family life. It is defined as the conflict that arises when demands from work and family roles are to some extent mutually incompatible (17). This concept has been broadened to include non-worktime roles and responsibilities other than family – as individuals without dependant children can still experience conflicts between private life and work (18). In a modern world, individuals often have a multitude of responsibilities, interests and social groups, all of which need to be balanced with work commitments. This is better captured by the term work–life interference (WLI) (19). While the relationship of work and non-work is bidirectional, this contribution focusses on work interfering with private life, and not vice versa since this direction is generally found to be stronger and more common (20).

A theory was previously proposed for why WLI could be an intermediate step on the causal pathway between WTC and subsequent health/well-being. If workers can self-determine working hours and regulate time to match individual needs and align them with family and other responsibilities, perceiving WLI should be less likely and work/non-work balance would be promoted (1). In turn, this balance facilitates maintaining well-being and good health-related behavior (21). In contrast, lower levels of WTC increase the likelihood to experience WLI while higher levels are associated with a reduction in WLI (22). Moreover, one study found differential effects between the two sub-dimensions of WTC. While control over time off was directly associated with WLI, control over daily hours only buffered against negative effects from long contractual working hours on WLI (23). High WLI is linked to unfavorable consequences for health, for example major depression (24), emotional exhaustion (25), headaches, and sleep problems (26). Moreover, ill-health can aggravate WLI and decrease WTC; research on reversed causal relationships is scarce but particularly mental health has been found to affect different work characteristics, either by changing perceptions of the respective factor or changing the job and work environment (27).

So far, only two studies have looked at the mediating role of WLI in the WTC-health relationship. In a natural experiment, researchers found that an increase in WTC at time 1 decreased interference of work with private life (time 2), which was (at the same time point) associated with longer sleep duration, better sleep quality, less emotional exhaustion, and slightly more frequent physical exercise (2). Drawing conclusions on mediation from this study however is problematic as the design was not fully longitudinal (28).

A more recent study based on a sample of workers in the healthcare sector concluded that WLI did play a mediating role in the relation between WTC and emotional exhaustion (29). The author also found evidence that emotional exhaustion mediated effects of WTC on WLI, meaning WTC affected emotional exhaustion which in turn influenced WLI. Although this study made an important contribution, it has limitations: a brief time span of one year, WTC being measured only at baseline, and a narrow set of covariates (age, gender and sleep time). The study did not allow for detecting effects of WTC that unfold over a longer period of time, effects on other health outcomes or in other population groups, nor did it sufficiently address confounding bias.

## Aims

To extend evidence from past research, the present study investigated whether WLI mediates the relationship between the two sub-dimensions of WTC (control over daily hours and control over time off) with depressive and musculoskeletal symptoms. Particularly, we were interested in potential differential results between mental and physical health outcomes as well as the two WTC sub-dimensions. Additionally, we aimed to assess reversed mediational effects, direct, and reversed direct effects in these relationships.

## Methods

### Study design and population

The data come from the Swedish Longitudinal Occupational Survey of Health (SLOSH), which is a biennial postal survey. An open cohort of participants, SLOSH is based on the 2003–2011 Swedish Work Environment Survey (SWES), which consisted of a sample of gainfully employed Swedish residents (aged 16–64 years). A full cohort profile can be found elsewhere (30). Different questionnaires are completed by those in paid work (≥30% full-time) and those who are temporarily or terminally outside of paid work. The sample is approximately representative in terms of gender and distribution of labor market sectors.

The present study sample is based on participants who responded to at least one SLOSH questionnaire (for those in work) between 2010–2016 with a total sample size of 26 804 (response rates 2010: 56.4%, N=11 525; 2012: 56.8%, N=9880; 2014: 52.6%, N=20 316; 2016: 50.9%, N=19 360). The Regional Research Ethics Board in Stockholm ethically approved both SLOSH (2012/373-31/5) and the present study (2014/696-31/5).

### Measures

*Work-time control*. A 5-item scale adapted from Ala-Mursula et al (5) measured perceived control over working hours, rated on a 5-point Likert scale from 1 (very little) to 5 (very much). Items differentiate between two sub-dimensions of WTC: control over daily hours (items regarding length and starting and ending time of work) and control over time off (items on taking breaks, running private errands, taking vacation/leave) (5, 6). A discussion of properties of the scale can be found elsewhere (6). We calculated means for each sub-dimension and each of the four waves between 2010–2016. Cronbach’s alphas for control over daily hours were 0.92 (2010), 0.93 (2012), 0.93 (2014) and 0.93 (2016), and for control over time off 0.75, 0.75, 0.77, and 0.77.

*Work–life interference*. WLI was measured four times between 2010–2016 using a 4-item scale (19), with items such as “I come home from work too tired to do things I would like to do”. Responses were rated as “not at all”, “rarely”, “sometimes”, “often” or “almost all the time”; means were calculated. Cronbach’s alphas were 0.89 (2010), 0.89 (2012), 0.90 (2014), and 0.91 (2016).

*Depressive symptoms*. A 6-item subscale of the Symptom Checklist (SCL-CD) measured core depressive symptoms with items regarding feeling blue, having no interest in things, feeling low in energy, worrying too much, blaming oneself, and perceiving everything as effortful. The scale’s validity and unidimensionality has been previously confirmed (31). Respondents rated how troublesome symptoms were during the last week from “not at all” (0) to “extremely” (4). Sum scores were calculated for each wave from 2010–2016. Cronbach’s alphas were 0.92 (2010), 0.91 (2012), 0.91 (2014), and 0.89 (2016).

*Musculoskeletal symptoms*. Between 2010–2016, respondents were asked if they had been diagnosed with or experienced a disease in their back, joints or muscles during the last two years. Responses were rated as “no”, “yes, but doesn’t affect my life”, “yes, affects my life a little”, and “yes, affects my life a lot”.

### Covariates

Covariate selection was led by theoretical considerations and previous knowledge on which variables were related to at least two of the main constructs. In a second step, covariates were selected based on directed acyclic graphs (DAG). Participant’s gender and socio-economic position (manual, lower-manual, and medium-to-high non-manual work) were available from register data throughout the study period. Self-reported data were used for the following covariates in 2010–2016: (i) age, (ii) highest level of education, (iii) shift-work status (defined as those regularly working shift/rostered hours in- or excluding nights or exclusively night hours), (iv) weekly working hours (<10–≥55 hours per week), (v)civil (cohabiting yes/no), and (vi) parental status (yes/no).

### Statistical analysis

Based on a path analysis model in structural equation modelling, we used cross-lagged panel models for mediation analyses which take stability and correlation of measurements over time into account. The two health outcomes – depressive and musculoskeletal symptoms – were analyzed separately to limit model complexity and allow for detecting differential effects. Data preparation was performed in SPSS Statistics for Windows, version 22.0 (IBM Corp, Armonk, NY, USA) while SEM was executed in Mplus 7 (32). Maximum likelihood estimates are reported and full information maximum likelihood (FIML) was utilized to reduce bias due to missing data (33).

To test for full longitudinal mediation, we used a step-wise analytic approach that was adapted from Cole & Maxwell (28) and Little (34, 35). It needs to be established first whether including mediational paths to a baseline model improves model fit and second, whether any other causal processes above and beyond the mediational pathways are important (such as reversed mediation, direct effects or reversed direct effects). Evidence of other directed pathways does not automatically contradict mediation – the purpose is rather to evaluate the relations between constructs (34). Lastly, robustness of the mediation model was tested by entering all relevant directed paths, allowing different lags between time points within constructs, and adding covariates into the model. Mediation pathways should remain significant in this final model. Regarding covariates, gender, age, and highest educational level were included as time-stable variables and correlated with each construct at the first time point. The remaining covariates (socio-economic position, shift-work status, weekly working hours, civil and parental status) were measured at the first and last time point and allowed to correlate with all constructs at the respective time (eg, civil status 1↔WTC1; civil status 4↔WTC4).

**Figure 1:**
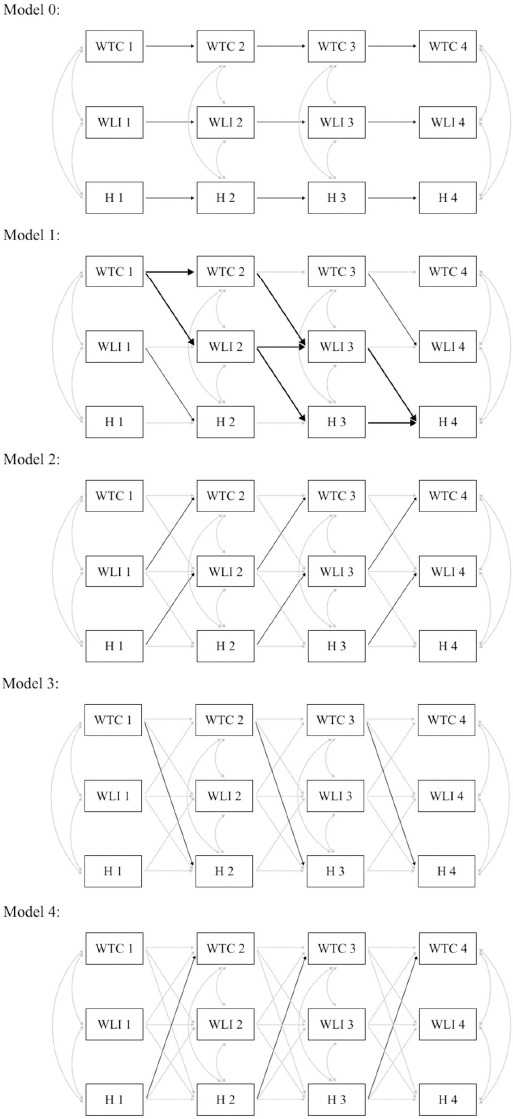
Exemplary longitudinal models compared against each other for work-time control (WTC), work-life interference (WLI) and health (H). Model 0 = auto-regressive paths and cross-sectional covariance between constructs; model 1 = causal mediation only (pathways for total indirect effect marked in bold); model 2 = additionally reversed causal mediation; model 3 = additionally direct effects; model 4 = additionally reversed direct effects.

In the modelling process, paths with the same time lag were held constant in all models, respectively (eg, WTC1→WTC2 was fixed to the same coefficient as WTC2→WTC3). Specifically, the following models (all of which including auto-regressive paths, eg, WTC1→WTC2) were estimated and compared against each other where applicable (figure 1): (i) a null-model (model 0) that allowed only cross-sectional covariance between constructs while omitting any cross-lagged relationships over time; (ii) a causal-mediation-only model (model 1) with additional cross-lagged pathways [eg, WTC1→WLI2→Health3 (H3)]; (iii) a full-mediation model (model 2) including causal mediational pathways *and* reversed mediational directions (eg, H1→WLI2→WTC3); (iv) a direct-effects model (model 3) where direct effects were additionally entered into the previous model (eg, WTC1→H2); (v) a reversed-direct effects model (model 4) where reversed directs effects were added (eg, H1→WTC2); and (vi) a final model (model 5), step-wise including directed paths that improved model fit in previous tests (eg, causal mediation, reversed mediation, direct and reversed direct effects), lags >1 within constructs, and covariates that was pruned (ie, non-significant paths removed). If mediation remained significant in this final model, the total indirect effect was calculated (based on a model letting other causal pathways be correlated) as the sum of the products (see figure 1, model 1) of all paths from WTC at the first time point to health at the last time point going through the mediator WLI (eg, WTC1→WLI2→H3→H4) (35). Significance/confidence intervals (CI) for the total indirect effect were assessed with bootstrap estimation (5000 samples).

Considering the amount of tests included in the analysis, the significance level was set to 0.001 for all tests (36). Models were evaluated both on model fit indices and the Chi^2^ statistics – the latter one needing to be treated cautiously as it is affected by large sample sizes and trivial disturbances easily become significant (37). Acceptable absolute fit is suggested by the root mean square error of approximation (RMSEA) <0.08 (38). Incremental fit is regarded as good with values >0.95 on the comparative fit index (CFI) (39). Relative model fit of nested models is indicated by lower values on the Bayesian information criterion (BIC) (40). Chi^2^ difference tests were used to compare nested models (model 0–4) with a significant result indicating that the reduced, simpler model may be too parsimonious and essential variables might be missing (28, 34).

## Results

### Descriptives

Sample characteristics are presented in table 1. Participants were on average 49 years old at baseline (SD=11.77). Lower levels of WTC (both sub-dimensions) were reported by those who were women, older, lower educated (particularly control over daily hours), single, without children, manual workers, fewer weekly working hours and working shift-work (particularly control over daily hours). WFC was experienced more often by those who were women, highly educated, single or without children (both small differences), non-manual workers, working more weekly hours, and working shifts. Those perceiving more depressive symptoms were women, older, better educated, without children, single, and working few or many weekly hours. More musculoskeletal symptoms were perceived by those who were women, younger, lower educated, cohabiting, working shift-work, manual workers, and working fewer weekly hours.

**Table 1:** Descriptive characteristics of the sample (N=26 804).

	2010		2016	
	
	N	%	N	%
Gender				
Male			11 959	44.6
Female			14 845	55.4
Highest educational level				
Primary/compulsory school (≤9 years)			3755	14.0
Secondary school/vocational training (≤11 years)			5763	21.5
Upper secondary school/gymnasium (≤13 years)			6362	23.7
University (<3 years)			3779	14.1
University (≥3 years)			7130	26.6
Civil status				
Living alone/single	2337	20.7	3988	20.9
Married/cohabiting	8945	79.3	15 086	79.1
Children living at home				
0	4768	42.3	6616	35.0
≥1 child	6514	57.7	12 265	65.0
Working time				
Any shift work/nights	1280	14.8	1782	13.7
Daytime/evening	7370	85.2	11 197	86.3
Weekly working hours/week				
≤35	1560	17.5	3014	22.5
36–45	5979	67.1	8217	61.0
46–55	1139	12.8	1850	13.8
>55	231	2.6	386	2.8
Occupation				
Manual workers	3675	32.7	5620	29.9
Lower non-manual workers	1655	14.7	2566	13.7
Medium-to-high non- manual workers	5558	49.4	10 372	55.2
Self-employed	356	3.2	222	1.2

Cross-sectional inter-correlations and means/standard deviations are found in the supplementary material (www.sjweh.fi/show_abstract.php?abstract_id=3887), table S1. Correlation coefficients of control over daily hours/time off varied between -0.03– -0.20 for WLI, -0.04– -0.14 for depressive symptoms, and -0.06– -0.12 for musculoskeletal symptoms. Coefficients for control over time off were generally larger than for control over daily hours. WLI correlated with depressive symptoms (0.33–0.51) and with musculoskeletal symptoms (0.10–0.16). All coefficients became in general smaller with a larger interval between measurements.

### Depressive symptoms

For control over daily hours we found improved model fit by adding causal mediational pathways via WLI (model 1, WTC→WLI β=-0.011, P<0.001; WLI→H β=0.647, P<0.001) to a model allowing only cross-sectional covariances (model 0). Entering reversed mediation pathways (model 2, H→WLI β=0.021, P<0.001; WLI→WTC β=-0.009, P=0.119) fit data better than causal mediation pathways only as indicated by model fit indexes and Chi^2^ difference tests. Adding direct (model 3, WTC→H β=-0.033, P=0.035) and reversed direct pathways (model 4, H→WTC β=-0.003, P=0.010) however did not substantially improve model fit (table 2). Thus, the final model (model 5, standardized estimates in supplementary figure S1) included causal mediation and initially also reversed mediation paths; since a part of the reversed mediation pathways became non-significant (WLI→WTC), these paths were pruned in model 5. Pathways from WTC to subsequent WLI were non-significant before inclusion of covariates (WTC→WLI β=-0.010, P=0.001). In the final model (including adjustment for potential confounders), all coefficients of causal mediation were significant (WTC→WLI β=-0.013, P<0.001, WLI→H β=0.391, P<0.001). The total indirect effect estimate was -0.018 (95% CI -0.026– -0.010, P<0.001, table 3); that is, for every 1-unit increase of the mean score of control over daily hours (range 1–5) a decrease in the sum score of depressive symptoms (range 0–24) of 0.018 is attributable to WLI.

**Table 2:** Model comparisons for control over daily hours/time off, work-life interference and depressive symptoms. [df=degrees of freedom; RMSEA=root mean square error of approximation; CFI=comparative fit index; BIC=Bayesian information criterion]. N=26 673.

	Control over daily hours						Control over time off					
	
	Chi^2^	df	RMSEA	CFI	BIC	Chi^2^ difference test (P-value)	Chi^2^	df	RMSEA	CFI	BIC	Chi^2^ difference test (P-value)
Model 0: Cross-sectional covariances	5516.793	60	0.058	0.904	587957		5954.752	60	0.061	0.901	562499	
Model 1: Causal mediation	4875.912	58	0.056	0.916	587336	vs. model 0 (<0.001)	5268.934	58	0.058	0.913	561834	vs. model 0 (<0.001)
Model 2: Reversed mediation	4455.549	56	0.054	0.923	586936	vs. model 1 (<0.001)	4847.476	56	0.057	0.920	561433	vs. model 1 (<0.001)
Model 3: Direct effects	4451.118	55	0.055	0.923	586942	vs. model 2 (0.035)	4843.496	55	0.057	0.920	561439	vs. model 2 (0.046)
Model 4: Reversed direct effects	4444.435	54	0.055	0.923	586946	vs. model 3 (0.010)	4833.062	54	0.058	0.920	561439	vs. model 3 (0.001)
Model 5: Final model	7221.791	209	0.035	0.935			5932.486	208	0.032	0.947		

**Table 3:** Standardized and unstandardized total indirect effect estimates.

	Total indirect effect estimates	

	Standardized	Unstandardized (95% CI)
Depressive symptoms		
Control over daily hours	-0.005	-0.018 (-0.026– -0.010)
Control over time off	-0.011	-0.053 (-0.065– -0.042)
Musculoskeletal symptoms		
Control over daily hours	-0.003	-0.003 (-0.004– -0.002)
Control over time off	-0.007	-0.007 (-0.008– -0.005)

For control over time off, causal mediational pathways (model 1, WTC→WLI β=-0.033, P<0.001, WLI→H β=0.639, P<0.001) fit data better than cross-sectional covariances only (model 0). Reversed mediation paths (model 2, H→WLI β=0.021, P<0.001, WLI→WTC β=-0.018, P<0.001) were retained in the final model (model 5) as those paths seemed to explain variance above and beyond causal mediational pathways. Direct effects (model 3, WTC→H β=-0.040, P=0.046) and reversed direct effects (model 4, H→WTC β=-0.003, P=0.001) did not substantially improve model fit (table 2). In the final model (supplementary figure S2), inclusion of covariates did not substantially change results and causal mediation pathways remained significant (WTC→WLI β=-0.035, P<0.001, WLI→H β=0.387, P<0.001). The total indirect effect was estimated at -0.053 (95% CI -0.065– -0.042, P<0.001), meaning that for every 1-unit increase in the mean score of control over time off, a decrease in the sum score of depressive symptoms of 0.053 is attributable to WLI.

### Musculoskeletal symptoms

For control over daily hours, model fit improved by entering causal mediation paths (model 1, WTC→WLI β=-0.013, P<0.001, WLI→H β=0.069, P<0.001) to the null model. Reversed mediation (model 2, H→WLI β=0.035, P=0.001, WLI→WTC β=-0.009, P=0.110), direct (model 3, WTC→H β=-0.025, P<0.001) and reversed direct paths (model 4, H→WTC β=-0.031, P<0.001) were retained in the final model (table 4). Covariate inclusion attenuated estimates only slightly. Reversed mediation paths from WLI to WTC became non-significant (thus were removed from the model), while causal mediation pathways from WTC to musculoskeletal symptoms via WLI remained significant (WTC→WLI β=-0.019, P<0.001, WLI→H β=0.053, P<0.001) in the final model (standardized estimates in supplementary figure S3). The total indirect effect was -0.003 (95% CI -0.004– -0.002, P<0.001).

**Table 4:** Model comparisons for control over daily hours/time off, work-life interference and musculoskeletal symptoms. [df=degrees of freedom; RMSEA=root mean square error of approximation; CFI=comparative fit index; BIC=Bayesian information criterion]. N=26 686.

	Control over daily hours						Control over time off					
	
	Chi^2^	df	RMSEA	CFI	BIC	Chi^2^ difference test (P-value)	Chi^2^	df	RMSEA	CFI	BIC	Chi^2^ difference test (P-value)
Model 0: Cross-sectional covariances	3700.171	60	0.048	0.921	405412		4218.627	60	0.051	0.915	380008	
Model 1: Causal mediation	3480.269	58	0.047	0.926	405213	vs. model 0 (<0.001)	3942.984	58	0.050	0.920	379753	vs. model 0 (<0.001)
Model 2: Reversed mediation	3424.078	56	0.047	0.927	405178	vs. model 1 (<0.001)	3885.440	56	0.051	0.921	379715	vs. model 1 (<0.001)
Model 3: Direct effects	3367.191	55	0.048	0.928	405131	vs. model 2 (<0.001)	3818.062	55	0.051	0.923	379659	vs. model 2 (<0.001)
Model 4: Reversed direct effects	3340.917	54	0.048	0.929	405115	vs. model 3 (<0.001)	3775.536	54	0.051	0.924	379626	vs. model 3 (<0.001)
Model 5: Final model	6503.789	205	0.034	0.935			5333.045	203	0.031	0.947		

For control over time off, model fit improved by entering causal mediation paths (model 1, WTC→WLI β=-0.036, P<0.001, WLI→H β=0.068, P<0.001) to the null model. Model fit further improved when adding reversed mediation (model 2, H→WLI β=0.030, P<0.001, WLI→WTC β=-0.018, P=0.110), direct (model 3, WTC→H β=-0.035, P<0.001), and reversed direct paths (model 4, H→WTC β=-0.030, P<0.001). Again, reversed indirect pathways from WLI to subsequent WTC became non-significant in the final model (model 5, supplementary figure S4) and were removed. Including covariates slightly attenuated most estimates, but causal mediation paths remained significant (WTC→WLI β=-0.045, P<0.001, WLI→H β=0.048, P<0.001). The total indirect effect was estimated at -0.007 (95% CI -0.008– -0.005, P<0.001).

## Discussion

Using a large, prospective sample of the Swedish workforce, this panel study found that WLI partially mediated effects of WTC (control over daily hours and time off) on two different health indicators: higher WTC led to less WLI, which in turn benefitted health to a small degree. Indirect effects were slightly larger for depressive symptoms and very small for musculoskeletal symptoms. These effects were stronger than those of other causal directions, with the exception of effects from depressive symptoms to subsequent WLI. Reversed mediation, direct, and reversed direct effects generally played a larger role for musculoskeletal compared to depressive symptoms. Out of the two sub-dimensions of WTC, control over time off consistently showed larger effects than control over daily hours.

Even though indirect effect estimates were significant but small-to-very small in size, this needs to be put in relation to the overall effect WTC has on WLI and health. In results presented in this study, cross-sectional correlations between concepts were very small but consistently significant. A comprehensive systematic review on the topic showed that effects of WTC on work–life balance were fairly small but evidence was deemed as strong and consistent (1). Evidence is slightly less consistent but increasing regarding health outcomes which may be due to lack of power to detect small effects in studies with smaller samples – larger studies tend to find effects (3, 12, 41) as opposed to smaller ones (42, 43). WTC is one small part of an individual’s psychosocial work environment and describes a very specific area. Therefore, small effect sizes are not unexpected and not unusual for other factors of the work environment either (44).

Results from this study are in line with previous research. Based on an occupational cohort with one year follow-up, a study found that WLI mediated the relationship between a general measure of WTC and emotional exhaustion (29). Our findings support these results but additionally highlight several points: (i) the two sub-dimensions of WTC differed consistently in size; (ii) indirect effect estimates varied for different health outcomes; (iii) reciprocal mediation, direct and reversed direct effects explained a smaller part of the effect; and (iv) effects remained robust even after controlling for a number of covariates.

### Sub-dimensions of WTC

In results presented here, control over time off (such as taking breaks and vacation) was associated more strongly with health indicators as well as WLI, and WLI better explained prospective effects of control over time off in contrast to control over daily hours. Previous research found that workers reported the highest need (even though prevalence was also high) for controlling when to take leave/vacation while control over daily hours was less required (and less common) (15). Self-determining daily starting and ending times of work might depend to a larger degree on cultural and organizational norms (45) and may therefore be less important to workers. Some research suggests that despite flexibility to determine starting and ending times of work, employees may not make the best choice in terms of recovery and sleep, particularly regarding shift-work (46). On the other side, control over time off may prevent WLI by allowing individuals to take breaks/run private errands when needed and to plan days off from work longer in advance.

In the literature, WTC is often used as one global measure (1) but the current study and previous research (3, 15) highlight that different aspects of WTC might affect health differently and vary in their mechanisms. Research on flexible working hours should take potential differential effects into account and consider measuring and analyzing sub-dimensions of WTC.

### Comparing the two health outcomes

We compared two health indicators regarding mental and physical health – depressive and musculoskeletal symptoms. Mediational pathways via WLI were stronger for depressive symptoms, particularly between WLI and depressive symptoms. This suggests that WTC may affect mental health to a larger extent than physical health. Few studies have directly compared mental and physical health regarding WTC. Findings from a Finnish study showed that while disability pension due to musculoskeletal disorders was more consistently associated with WTC, effects on disability pension due to mental disorders were stronger (with a smaller sample and wider confidence intervals) (12). Moreover, WLI may be more strongly related to mental health (26) and hence mediate effects of WTC on health more for mental than physical health. Other mediating variables might play a larger role in explaining effects of WTC on physical health, as indicated by the presence of other causal directions.

### Other causal directions

Even though causal mediation coefficients (WTC→WLI and WLI→H) were an important contributor to explained variance, we still found evidence for reversed mediational effects: health affected subsequent levels of WLI, which in turn influenced perceived WTC. We found similar results in a previous study where, although reciprocal effects were found, causal effects from WTC to subsequent depressive symptoms explained data better (3). Our results extend these findings and suggest that while WLI mostly acts as mediating variable in effects of WTC on health, there are some reversed causal processes: sub-optimal health increases perceived conflict between work and private life, which in turn decreases *ratings* of WTC. However, in all final models, we found that pathways from WLI to subsequent levels of WTC became non-significant, indicating that reversed effects were predominantly from health to subsequent WLI. Pathways from depressive symptoms to WLI were particularly strong. These changes could be “objective” decreases in WLI, but it is more likely that a decrease in health changes perceptions of stressors and resources, which in turn affects rating of WLI.

In our results, direct pathways explained part of the effect from WTC to subsequent musculoskeletal symptoms but did not substantially improve model fit regarding depressive symptoms. This means either that WTC directly affects physical health – eg, by promoting physical relaxation or facilitating healthy behavior – or, more likely, that other mediating variables play an important role within the effect chain (summarized as the direct effect in our models), eg, a sense of autonomy, reduced stress, increased muscular relaxation and reduced physical strain. In line with the effort–recovery model (16), recovery may be of particular interest to investigate as potential mediator. WTC allows workers both to recover (mentally and physically) by taking breaks when needed at work and to schedule time off from work. If workers utilize WTC, especially control over time off, to increase recovery opportunities, this could buffer against work overload and chronic physical strain but also WLI, fatigue and other negative health effects.

In our study, direct and reversed direct effects played a smaller role for depressive symptoms. This might indicate that WLI is a more important link in the chain of causation for depressive symptoms, meaning that more of the effect from WTC to depression goes via WLI than for musculoskeletal symptoms. This notion is supported by results from Hämmig et al (26) showing that work–life conflict was more strongly associated with mental than physical health. Reversed direct effects (but also reversed mediational paths) could indicate that results regarding musculoskeletal symptoms may be slightly biased by unmeasured symptoms of health before the study. Overall, our results suggest the relationship between WTC and musculoskeletal disorders is less clear, and WLI seems to explain a smaller part of this effect.

### Strengths and limitations

The present study has some key strengths. We used a prospective design with panel data spanning six years, which is approximately representative of the Swedish working population. The analysis accounted for stability and correlation of measurements over time of all variables. We examined two different health indicators and considered a number of covariates.

However, several limitations need to be highlighted. This study is almost exclusively based on self-reported data and known issues with this type of data apply. As with all observational studies, we cannot rule out that unmeasured confounding and especially intermediate confounders could have biased mediational estimates. To minimize validity concerns, we utilized DAG and included several relevant covariates, but estimates might still be biased due to not-included variables. The statistical method used here comes with strong assumptions, among them the one of ergodicity, which allows to generalize from population processes to the individual (47). While especially WTC may be less affected by stable, trait-like differences, mixing within- and between-person variance may still have created bias in our results (specifically considering reversed causation). Attrition could have been a problem in our study. As a number of baseline data are available for non-responders after the first included wave, it was possible to utilize FIML estimation to fill in missingness under the missing at random (MAR) assumption (48). Musculoskeletal symptoms were measured with one categorical item only. We repeated analyses with diagonally weighted least squares (WLSMV) estimation and results did not differ to maximum likelihood estimation regarding model decisions and general direction. Longitudinal mediation analysis relies heavily on using data with an optimal lag between time points, often without knowing what the optimal lag is for effects to fully unfold (34). In our study, repeated measurements were available every other year allowing us to examine potential effects that develop over a longer period of time and become manifest in mental and physical health. However, we cannot be certain that two years is the optimal lag to reach maximum effect size and we were unable to investigate effects unfolding over a shorter (or longer) period of time.

### Practical implications

Even though effects of WTC via WLI on subsequent health were comparably small, results presented here have implications for employers. Addressing factors of work environment can be useful as it reaches the majority of, if not all employees with an intervention. As WTC is a modifiable factor in most occupations (at least to some degree), an increase in autonomy regarding working hours could help employees to align work and private life better and lessen build-up of health problems, particularly regarding mental health. Especially control over time off appears to buffer against work–life conflict, which in turn can prevent ill-health. At the same time, our results suggest that ill-health, especially mental health issues, can negatively impact levels of perceived WLI and WTC. If baseline health within a group of workers is already deteriorating, it is most likely helpful to not only increase control over working hours but also address health issues which in turn may improve perceived WLI and WTC.

### Concluding remarks

We found evidence that WLI partially mediates effects from WTC to subsequent health – particularly regarding depressive symptoms and to a lesser degree for musculoskeletal pains. Our results highlight that one sub-dimension of WTC (ie, control over time off) seemed to buffer more against WLI and in turn was associated with a decrease in depressive and musculoskeletal symptoms. Reversed mediational and direct effects still played a role, indicating both reversed causality and remaining unexplained mechanisms, especially regarding physical health. Future research needs to further advance our understanding of the causal pathway between WTC and long-term health effects and different potential mediators should be investigated.

## Supplementary material

Supplementary material
