# Supplementary material for: The mediating effect of work–life interference on the relationship between work-time control and depressive and musculoskeletal symptoms
Source: Scand J Work Environ Health. 2020 Jan 30;46(5):469–79. doi: 10.5271/sjweh.3887 (PMC7737796; doi:10.5271/sjweh.3887)
Supplement: Supplementary material [file SJWEH-46-469-S001.pdf]

The mediating effect of work-life interference on the relationship between work-time control and depressive and musculoskeletal symptoms [1]

by Sophie C Albrecht, MSc,[2] Göran Kecklund, PhD, Constanze Leineweber, PhD

1. *Supplementary table and figures*
2. *Correspondence to: Sophie C Albrecht, Stress Research Institute, Stockholm University, 106 91 Stockholm, Sweden. [E-mail: sophie.albrecht@su.se]*

Supplementary table S1: Estimated means, standard deviations (SD) and inter-correlation between control over daily hours and time off (scale from 1 to 5), work-life interference (1 to 5), depressive symptoms (0 to 4) and musculoskeletal symptoms (1 to 4); correlations are significant with p<0.001 unless marked with ns=not significant.

Variable	Mean	SD	1	2	3	4	5	6	7	8	9	10	11	12
1 Control over daily hours 2010	2.832	1.343	-											
2 Control over daily hours 2012	2.817	1.332	0.728	-										
3 Control over daily hours 2014	2.871	1.377	0.689	0.755	-									

4 Control over daily hours 2016	2.889	1.363	0.647	0.711	0.735	-								
5 Control over time off 2010	3.143	1.041	0.699	0.604	0.579	0.575	-							
6 Control over time off 2012	3.137	1.031	0.589	0.712	0.609	0.600	0.738	-						
7 Control over time off 2014	3.180	1.062	0.577	0.635	0.721	0.620	0.714	0.763	-					
8 Control over time off 2016	3.202	1.055	0.567	0.608	0.617	0.722	0.703	0.738	0.761	-				
9 WLI 2010	2.680	0.945	-0.059	-0.048	-0.034 (ns)	-0.030	-0.170	-0.143	-0.126	-0.121	-			
10 WLI 2012	2.561	0.931	-0.042 (ns)	-0.082	-0.049 (ns)	-0.048	-0.141	-0.188	-0.156	-0.148	0.667	-		
11 WLI 2014	2.665	0.990	-0.055	-0.093	-0.100	-0.075	-0.153	-0.184	-0.201	-0.164	0.598	0.679	-	
12 WLI 2016	2.660	0.995	-0.069	-0.094	-0.078	-0.108	-0.145	-0.165	-0.166	-0.209	0.545	0.599	0.665	-
13 Depressive symptoms 2010	5.164	5.175	-0.055	-0.054	-0.044 (ns)	-0.061	-0.107	-0.097	-0.090	-0.104	0.490	0.404	0.374	0.358
14 Depressive symptoms 2012	4.501	4.846	-0.035 (ns)	-0.065	-0.049 (ns)	-0.072	-0.081	-0.120	-0.102	-0.111	0.378	0.498	0.393	0.381

15 Depressive symptoms 2014	4.996	4.980	-0.053	-0.068	-0.071	-0.063	-0.093	-0.118	-0.124	-0.117	0.353	0.408	0.520	0.412
16 Depressive symptoms 2016	4.863	5.003	-0.039 (ns)	-0.058	-0.058	-0.070	-0.084	-0.091	-0.090	-0.126	0.328	0.363	0.378	0.514
17 Musculoskeletal symptoms 2010	1.576	0.961	-0.076	-0.063	-0.064	-0.062	-0.093	-0.083	-0.091	-0.091	0.128	0.136	0.127	0.103
18 Musculoskeletal symptoms 2012	1.597	0.981	-0.076	-0.079	-0.089	-0.081	-0.101	-0.103	-0.101	-0.108	0.147	0.163	0.154	0.125
19 Musculoskeletal symptoms 2014	1.605	0.994	-0.072	-0.072	-0.093	-0.084	-0.086	-0.092	-0.104	-0.104	0.140	0.154	0.164	0.130
20 Musculoskeletal symptoms 2016	1.602	0.998	-0.079	-0.094	-0.091	-0.092	-0.095	-0.115	-0.103	-0.115	0.146	0.156	0.153	0.141

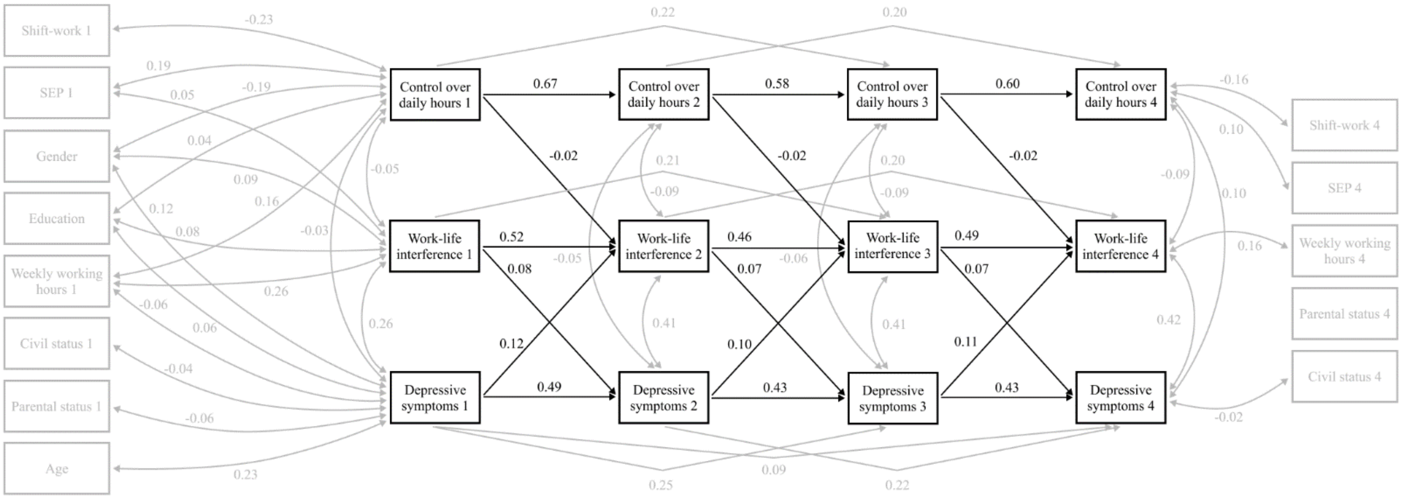

Supplementary Figure S1: Final model (Model 5) and standardized coefficients for control over daily hours, work-life interference and depressive symptoms (correlations between covariates not displayed).

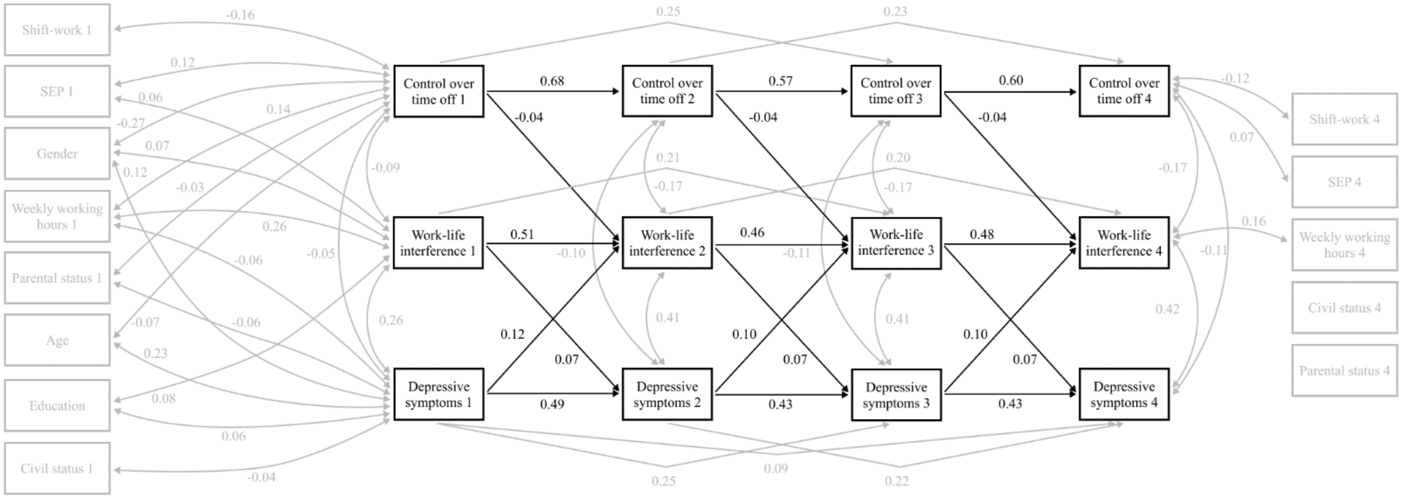

Supplementary figure S2: Final model (Model 5) and standardized coefficients for control over time off, work-life interference and depressive symptoms (correlations between covariates not displayed).

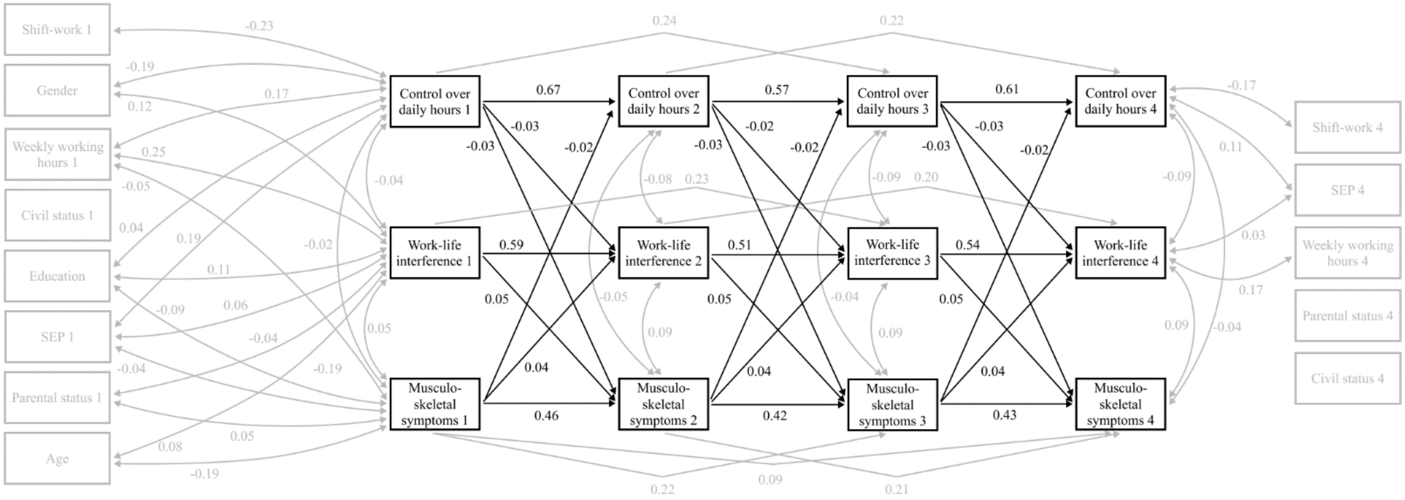

Supplementary figure S3: Final model (Model 5) and standardized coefficients for control over daily hours, work-life interference and musculoskeletal symptoms (correlations between covariates not displayed).

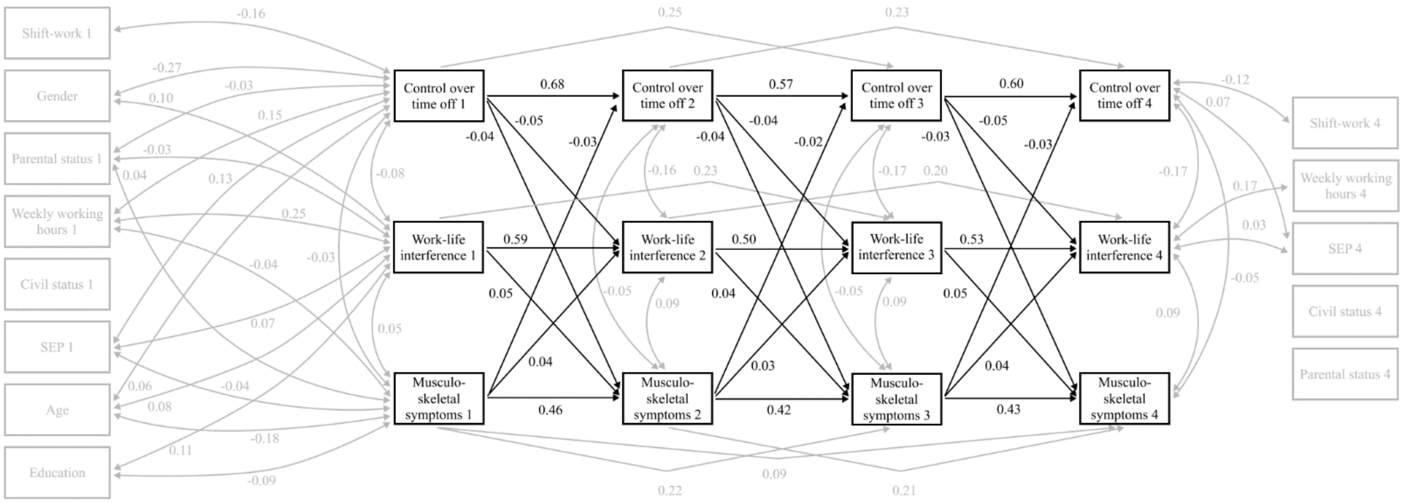

Supplementary figure S4: Final model (Model 5) and standardized coefficients for control over time off, work-life interference and musculoskeletal symptoms (correlations between covariates not displayed).
